# Conformal Growth of Stable 1T-Phase WS_2_ by Alkali Metal-Halide-Assisted Chemical Vapor Deposition

**DOI:** 10.1021/acs.cgd.6c00304

**Published:** 2026-07-18

**Authors:** Alessia Motta, Andrea Serafini, Paolo Targa, Davide Codegoni, Carlo Grazianetti, Christian Martella

**Affiliations:** † 9327 CNR IMM, Unit of Agrate Brianza, Via C. Olivetti 2, Agrate Brianza I-20864, Italy; ‡ 68232 STMicroelectronics, Via C. Olivetti 2, Agrate Brianza I-20864, Italy

## Abstract

The potential exploitation of two-dimensional transition metal dichalcogenides (TMDs) like WS_2_ in nanotechnology applications still require the development of efficient synthesis protocols for their large-scale growth as well as their polymorphic phase selection. Indeed, in the former framework, the growth protocols developed for chemical vapor deposition (CVD) typically use seeding promoters and/or salts (e.g., alkali metal halides) to enhance the adhesion to the substrate and lower the temperature for metal oxide precursor (e.g., WO_3_) dissociation. In the latter framework, the wealth of electronic properties of WS_2_ make it semiconducting or metallic depending on the crystallographic phase. In this work, we use potassium chloride-assisted CVD to produce the metastable 1T phase of WS_2_ without the need of additional seeding promoter even on patterned substrates demonstrating conformal and stable (over months and up to 200 °C) deposition. Furthermore, thermal Raman spectroscopy measurements were carried out determining the temperature coefficients, even for the specific 1T-phase sensitive Raman mode. Our findings demonstrate that the chemistry of salt-assisted CVD growth process can be exploited for TMDs phase filtration without constraints on the large-scale and conformal growth.

## Graphical abstract

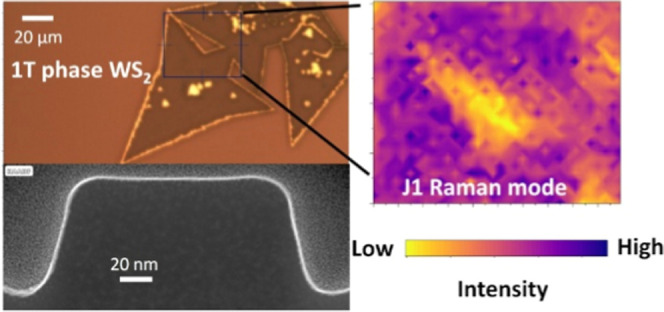

## Introduction

In recent years, two-dimensional (2D) transition-metal dichalcogenides (TMDs) have garnered significant attention due to their unique tunable optical and electrical properties, which make them suitable for next-generation technologies. Among these, tungsten disulfide (WS_2_) stands out for its high carrier mobility, strong photoluminescence,^1,2^ and excellent chemical and thermal stability.^3,4^ These features position WS_2_ as a promising candidate for applications in optoelectronics,^5,6^ catalysis,^7,8^ and flexible electronic devices.^9^

WS_2_ exhibits several polymorphic phases, each with distinct structural and electronic properties. The most common is the semiconducting 1H phase, characterized by a trigonal prismatic coordination where sulfur atoms are vertically aligned along the *c*-axis, forming a hexagonal symmetry around the tungsten atoms. A lateral shift of one sulfur plane transforms this structure into the 1T phase, which adopts octahedral coordination and displays metallic behavior. A simplified schematic structure of the sulfur (S) and tungsten (W) coordination in the two polymorphic phases is reported in Figure 1a. A further structural distortion results in the 1T′ phase, a semiconducting polymorph with distorted octahedral coordination and tetragonal symmetry.

**Figure 1:**
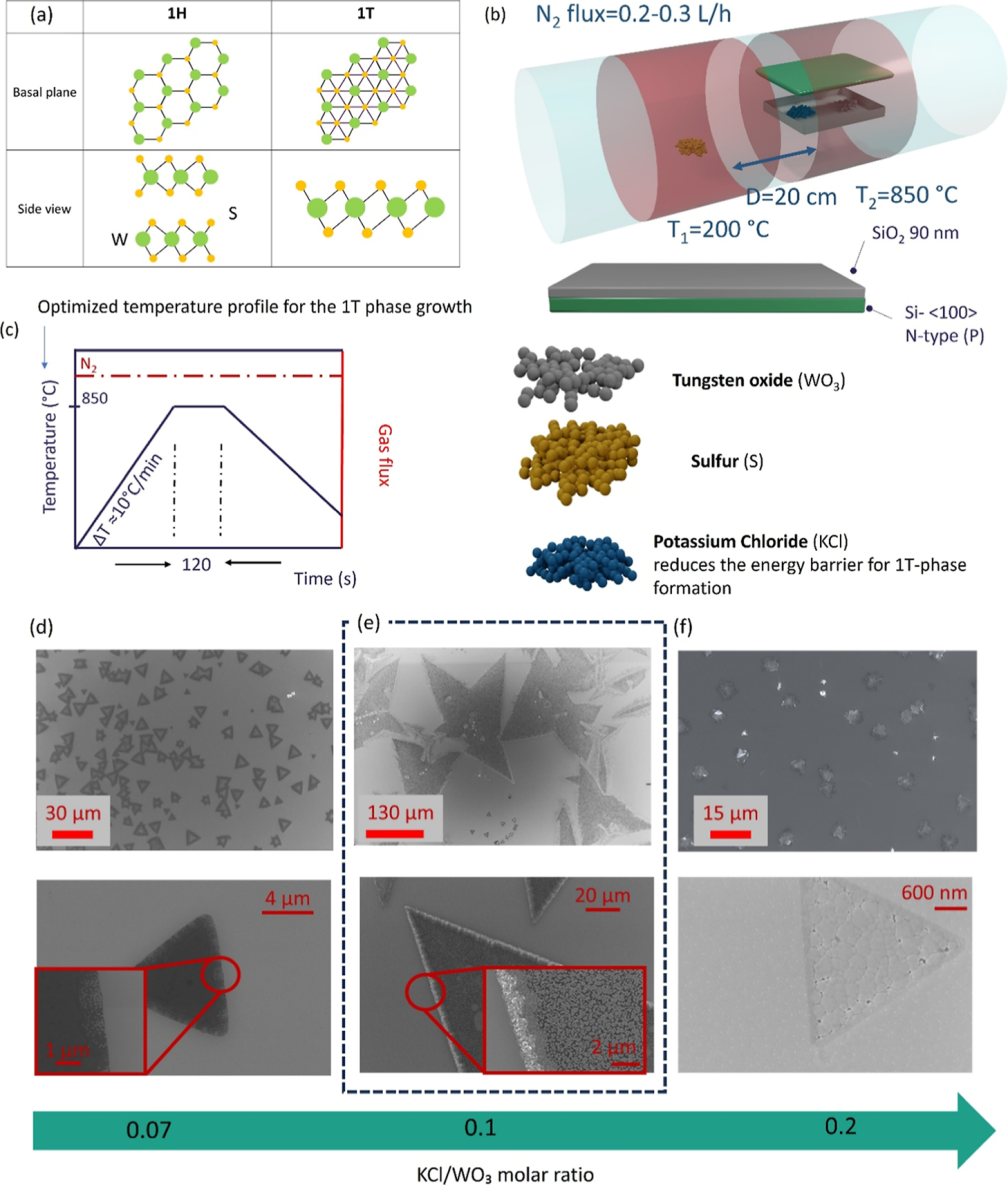
(a) Sketch of the 1H (left) and 1T (right) phases of WS_2_. (b) Cartoon of the alkali metal halide chemical vapor deposition (CVD) process for the WS_2_ synthesis. (c) Thermal ramp of the CVD process. Scanning electron microscopy (SEM) images of monolayer WS_2_ flakes synthesized with different KCl/WO_3_ ratios: (I) 0.07 (d), (II) 0.1 (e), and (III) 0.2 (f). Figure (d,e) also feature an inset highlighting the edges of a flake.

The 1T phase has a relative energy of approximately 0.9 eV higher than that of the 1H phase, which is the most thermodynamically stable,^10^ making it metastable under ambient conditions and more difficult to produce.^11^ Despite this, the different WS_2_ phases can be changed through intralayer atomic displacements, and phase transitions can be induced by various external stimuli, including thermal treatment,^12^ laser irradiation,^13^ electron beam exposure,^14^ strain,^15^ and chemical doping.^16^ Notably, the 1T-to-1H transformation can occur spontaneously at room temperature over time and is accelerated by thermal annealing.^17^ The 1T-phase of WS_2_, and in general of 1T-phase TMDs, attract attention due to its potential in advanced applications like superconductivity,^18^ ferroelectricity,^19^ and energy technologies.^20^ However, the 1T-phase of WS_2_ scalable synthesis remains challenging since traditional methods based on chemical exfoliation offer limited control over layer thickness and phase purity, thus limiting practical integration.

Atomic layer deposition (ALD) and pulsed laser deposition (PLD) have emerged as effective techniques for the synthesis of WS_2_ thin films. In particular, ALD enables wafer-scale growth with excellent control over thickness, uniformity, and tunable morphology and doping, making it highly suitable for device integration.^21^ Similarly, PLD has been demonstrated as a viable route for the direct synthesis of few-layer WS_2_ with good structural quality and tunable phase composition.^22^ However, the direct growth of the metastable 1T phase remains challenging due to the high energy barrier associated with 1T-WS_2_ formation.^23^ In this context, both ALD and PLD have so far struggled to achieve large-scale growth of a pure 1T phase, typically yielding mixed 1H–1T structures instead. CVD, on the other hand, has emerged as a promising approach for phase-selective growth over large areas.^24,25^ For instance, large-area 1T-WS_2_ films have been obtained via plasma-enhanced CVD through the sulfurization of predeposited W thin films in sulfur-rich environments.^26^

Nevertheless, such sulfurization processes involve heterogeneous solid–vapor reactions, causing the resulting WS_2_ films to inherit the morphological and structural features of the initial W layer, often with limited crystalline quality. More recently, advances in precursor design and optimization of CVD growth parameters have demonstrated the possibility of directly synthesizing well-defined crystalline phases, including the metallic 1T phase.^11,27^

For instance, the incorporation of alkali metal halides such as sodium chloride (NaCl) or potassium chloride (KCl) has been shown to significantly lower the energy barrier for 1T-phase formation. To date, the successful growth of 1T-WS_2_ monolayers has been reported by Li et al. through controlled tuning of the WO_3_/NaCl ratio,^27^ and by Lin et al., who employed a combination of NaCl and Fe_2_O_3_ as the halide source and catalytic metal oxide, respectively.^11^ While Li et al. achieved selective formation of the 1T phase, by finely tuning of the salt ratio, large-area was not fully addressed, leaving room for further investigation. In contrast, Lin et al. developed a process enabling the simultaneous growth of 1H and 1T-WS_2_ phase, in butterfly-like flakes. This phase coexistence was likely due to the inhomogeneous halide distribution across the substrate surface, which complicates precise control over the phase purity. In addition to the “phase filtration”, i.e., the possibility of select the WS_2_ phase on-demand, nowadays the capability to grow a specific TMD’s phase on prepatterned substrates to shape engineering its properties is of paramount importance.^28^ In this work, we present a method for synthesizing monolayer 1T-WS_2_ using salt-assisted CVD (Figure 1b). By introducing KCl, we drastically reduce the formation energy of the 1T phase. Moreover, we exploit KCl-assisted CVD to demonstrate its dual mechanisms in WS_2_ crystal growth. First, KCl catalyzes WO_3_ decomposition via volatile tungsten oxyhalides (WO_2_Cl_2_ and WOCl_4_), markedly enhancing the W adatom flux for nucleation and growth in an alkali metal-halide-assisted CVD. Second, KCl serves as a surface promoter, boosting adatom mobility and flake-substrate adhesion, which circumvents limitations of using pure WO_3_ precursors, such as sparse coverage and poor interfacial contact of the flakes on SiO_2_ substrates. To study flake-substrate adhesion, we employ prepatterned substrates to rigorously probe KCl-mediated adhesion through cross-sectional transmission electron microscopy (TEM), revealing intimate monolayer 1T-WS_2_-substrate contact even at pronounced spatial curvatures (e.g., trench edges). Finally, we use thermal Raman spectroscopy to validate the thermal stability and determine the thermal coefficients of the 1T-phase WS_2_ up to 200 °C.

## Experimental Section

### Material Synthesis

WS_2_ crystal synthesis was performed using a CVD system equipped with a hot-wall furnace and a 1-in. diameter fused quartz tube. In Figure 1, a schematic representation of the system (Figure 1b) and its associated processes (Figure 1c) have been provided to facilitate comprehension. A fused silica boat containing a mixture of WO_3_ powder (99.9%, Fluka) and KCl powder (>90%, Sigma-Aldrich) was positioned at the center of the quartz tube. The substrates, consisting of 90 nm-thick SiO_2_ on n-doped Si, were placed approximately 15 mm above the precursor powders, with the polished side facing downward. A separate boat containing sulfur powder (99.98%, Merck) was placed in the upstream zone of the tube. Prior to growth, the substrates underwent a three-step cleaning process: ultrasonic bath in acetone for 5 min, isopropanol bath for 5 min, and rinse in deionized water for 5 min. After cleaning, the substrates were dried using a nitrogen gas stream. Surface functionalization was then carried out via oxygen plasma treatment for 10 min at 500 W. For the growth process, the furnace was ramped up to 850 °C at a rate of 10 °C/min and maintained at that temperature for 20 min under a continuous nitrogen flow.

### Patterning of the Substrate

The nanoscale patterns were fabricated by ultraviolet (UV) optical lithography generating a photoresist mask and then dry etching (reactive ion etching) in order to transfer the pattern into the SiO_2_ without reaching the underlying silicon substrate.

### Scanning Electron Microscopy

The morphology of WS_2_ layers was characterized by field-emission SEM (FE-SEM, SUPRA 40, Zeiss) using an in-lens detector and an acceleration voltage of 15 kV.

### Micro-Raman Spectroscopy

The vibrational properties of the deposited sample were verified by Raman spectroscopy in z-backscattering geometry using a Renishaw spectrometer (In-Via) equipped with a solid-state laser source of excitation wavelength 514 nm/2.41 eV. The laser source was coupled with an optical microscope and objective with numerical aperture 0.75 and magnification 50×. The laser power on the sample was kept below 5 mW to avoid sample damage. A probe station (Linkam model HFSX350) equipped with a thermal stage was used to conduct Raman investigation varying the temperature of the sample in the range 30–200 °C. The Raman spectra were fitted using a sum of Lorentzian functions to extract the peak position (Xc) and full width at half-maximum (fwhm) for each vibrational mode.

### Scanning Transmission Electron Microscopy

The samples were extensively investigated utilizing STEM-related techniques such as dark-field STEM (DF-STEM) for the microstructural characterization, while the chemical properties were investigated by energy-dispersive X-ray spectroscopy (EDX). DF-STEM and EDX analyses were carried out on electron-transparent lamella obtained using focused ion beam (FIB) thinning of cross-section TEM lamellae. The lamellae preparation was performed using a Thermofisher Helios G5UX FIB. In all cases, particular care was taken to limit the heating and ballistic effects of ion irradiation on the samples during the final ion milling steps. In particular, a new advanced approach has been applied, reducing currents and energies progressively from a value of 30 keV to a value of low keV in order to avoid material amorphization and damage. The STEM images were performed with a Thermofisher Themis Z G3 aberration-corrected scanning transmission electron microscope equipped with an electron gun monochromator operating at 200 kV acceleration voltage. To limit the electron beam damage, all the STEM and EDX images were acquired with a low beam current (0.5 nA). The elemental maps were obtained with a step size of 8 Å, and the data were processed using the Gatan GMS Digital Micrograph 3.23 software.

## Experimental Results

### KCl/WO_3_-Dependent Growth

The concentration of alkali metal halides plays a crucial role in the CVD growth of TMDs, as it promotes the formation of volatile metal-oxyhalide species and alkali-metal-containing ternary intermediates with reduced melting points.^29,30^ In this study, we systematically investigated the salt ratio (KCl/WO_3_, defined as the mass ratio between the alkali metal halide and metal oxide precursor) to achieve large-area, uniform 1T-phase WS_2_ monolayers. We identified three conditions for KCl/WO_3_ molar ratios as low (I) 0.07, intermediate (II) 0.1, and high (III) 0.2 to represent a carefully designed exploration of the salt–promotion parameter space in alkali-metal halide-assisted CVD.

Figure 1d–f shows SEM images of WS_2_ flakes at the different and increasing salt-precursor molar ratios. Figure 1d shows the formation of triangular flakes obtained for molar ratio KCl/WO_3_ = 0.07 (case I). At this low salt concentration, the growth remains relatively close to classical CVD based on pure WO_3_ precursor, with only marginal formation of oxyhalide species. Under this condition, the density of flakes on the surface is low and the flakes’ lateral size is in the range of ∼10 μm (see Figure 1d). We further note that minor imperfections are visible, as reported in the inset of Figure 1d, along the flake edges in the form of bright spots, which are likely related to salt byproduct clustering. Figure 1e shows SEM images of crystal flakes grown at a KCl/WO_3_ molar ratio of 0.1 (case II). At this intermediate salt concentration, both the surface coverage and the lateral dimensions of the flakes increase, reaching lateral size exceeding 100 μm. As for the previous case, the formation of clusters is localized at the edges of the crystal. Increasing the salt concentration up to a molar ratio KCl/WO_3_ = 0.2 (case III) has dramatic effects on the growth. SEM investigations shown in Figure 1f reveal that, at such elevated salt concentration, the lateral size of the flakes is drastically reduced <2 μm along with their spatial density on the substrate surface. More importantly, the clusters, previously localized only at the flake edges (Figure 1e), coalesce into a continuous coating across the flake surface as reported in Figure 1f. This increase in the cluster coverage can be related to the increase of tungsten oxyhalide formation (WO_2_Cl_2_, WOCl_4_) at the KCl/WO_3_ molar ratio of 0.2 and might affect the electronic properties of WS2. From the comparative study of the three different cases, we can rationalize the core principle of salt-assisted CVD: alkali metal halides such as KCl dramatically lower the sublimation temperature of WO_3_ by forming volatile tungsten oxyhalides (e.g., WO_2_Cl_2_ and WOCl_4_), which are absent in pure oxide precursor systems. The promoter-to-precursor molar ratio directly modulates the concentration of these volatile intermediates, thereby controlling the flux of W adatoms to the substrate surface and enabling the nucleation of many larger flakes.

We performed Raman spectroscopy to unambiguously identify the formation of WS_2_ and its crystal phase. Figure 2a shows the main Raman modes observed in WS_2_ and their fit using Lorentzian functions. Figure 2b shows the Raman spectra of WS_2_ flakes synthesized by CVD using different KCl/WO_3_ ratios equal to 0.07 (light green), 0.1 (green), and 0.2 (blue line). The Raman spectra display the characteristic vibrational modes of WS_2_, particularly the A_1g_ mode, associated with out-of-plane vibrations of sulfur atoms, located around 417 cm^–1^, and the combined 2LA + E^1^_2g_ modes. The E^1^_2g_ mode, corresponding to in-plane vibrations of tungsten and sulfur atoms in opposite directions, appears ∼356 cm^–1^, while the 2LA mode is observed at ∼350 cm^–1^.^31^

**Figure 2:**
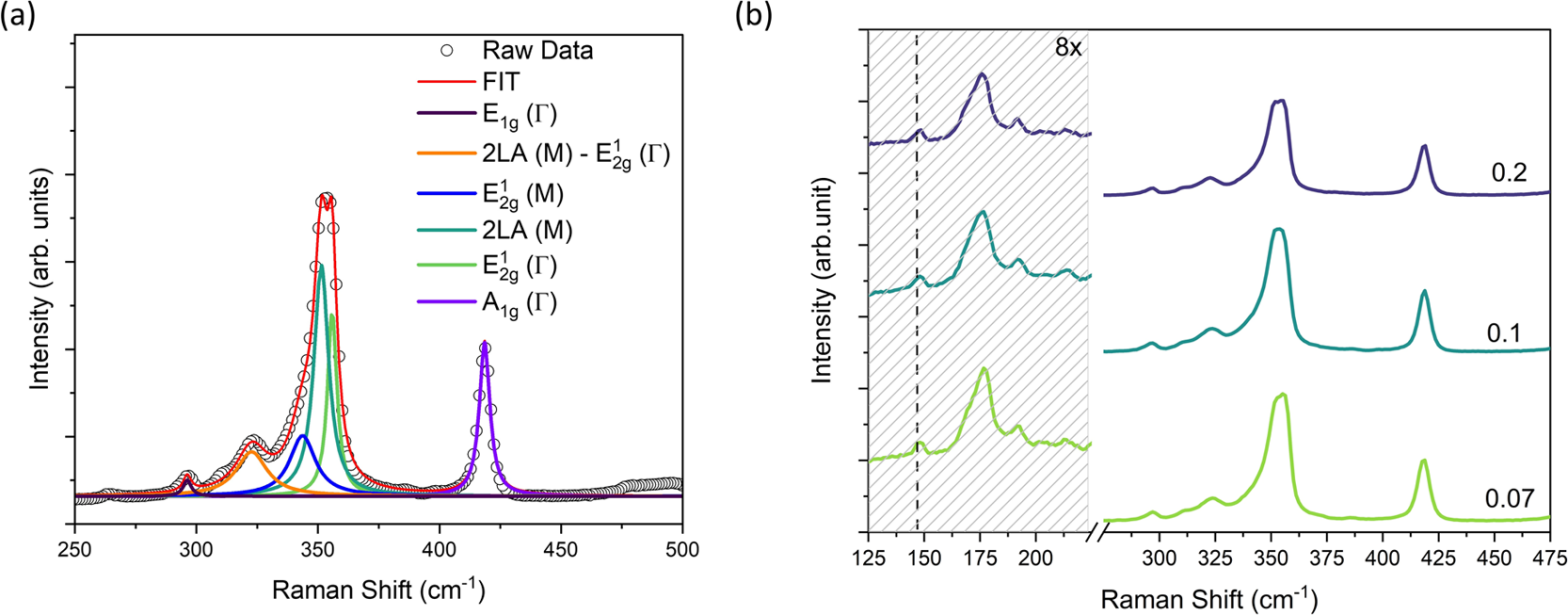
(a) Example of the deconvolution of the WS_2_ Raman spectrum using Lorentzian components. (b) Raman spectra from different monolayer flakes grown varying the KCl/WO_3_ ratios (0.07, 0.1, and 0.2). In the highlighted low-frequency region, the Raman spectra were enhanced by *a* factor of 8 to better display the J_1_ mode at 146.7 cm^–1^.

The frequency difference between the E^1^_2g_ and A_1g_ modes and the intensity ratio between the 2LA and E^1^_2g_ modes were used to evaluate the layer thickness.^1,30,31^ Specifically, the measured values for the E^1^_2g_–A_1g_ separation and 2LA/E^1^_2g_ intensity ratio were respectively: 62.1 ± 0.6 cm^–1^ and 1.3 ± 0.1 for case I, 62.9 ± 0.6 cm^–1^ and 1.3 ± 0.2 for case II, and 62.6 ± 0.1 cm^–1^ and 1.3 ± 0.1 for case III. To gain deeper insight into the flakes’ crystal structure, the low frequency Raman region between 120 and 230 cm^–1^ is investigated and reported (magnified by a factor of 8) in Figure 2a. In this region, we observe the presence of the LA­(M) mode at ∼175.7 cm^–1^, which is commonly used as a fingerprint of defect-induced activation,^27^ and the J_1_ mode at 146.7 cm^–1^, which is attributed to longitudinal vibrations of the S–W–S bonds and has been associated with the 1T metallic phase of WS_2_.^32^ The presence of the J_1_ mode even at the lowest KCl/WO_3_ ratio demonstrates the role of the alkali metal-halide-assisted process in stabilizing the 1T-WS_2_ phase as reported in refs 27, 11 , using NaCl salt. By analogy, K^+^ ions can have a similar role to Na^+^, acting as n-type dopants that donate electrons to the system and thereby stabilize the 1T phase. By combining the topographical images by SEM and the Raman spectroscopy characterization, the 0.1 molar ratio (case II) provides the best conditions to achieve large flakes of 1T-phase WS_2_.

#### Conformal Growth on Patterned Samples

Aiming at tailoring the shape of the so-grown WS_2_ flakes to tune their properties,^28^ we investigated the alkali metal halide-assisted CVD growth on a patterned sample as illustrated in Figure 3. The patterned substrate is a SiO_2_ wafer with an array of trenches having lateral width, *w* = 170 nm, and height, *h* = 120 nm, and the period of the array is *P* = 2 w. In light of the experiments conducted on flat surfaces, a 0.1 salt ratio concentration (case II) was selected.

**Figure 3:**
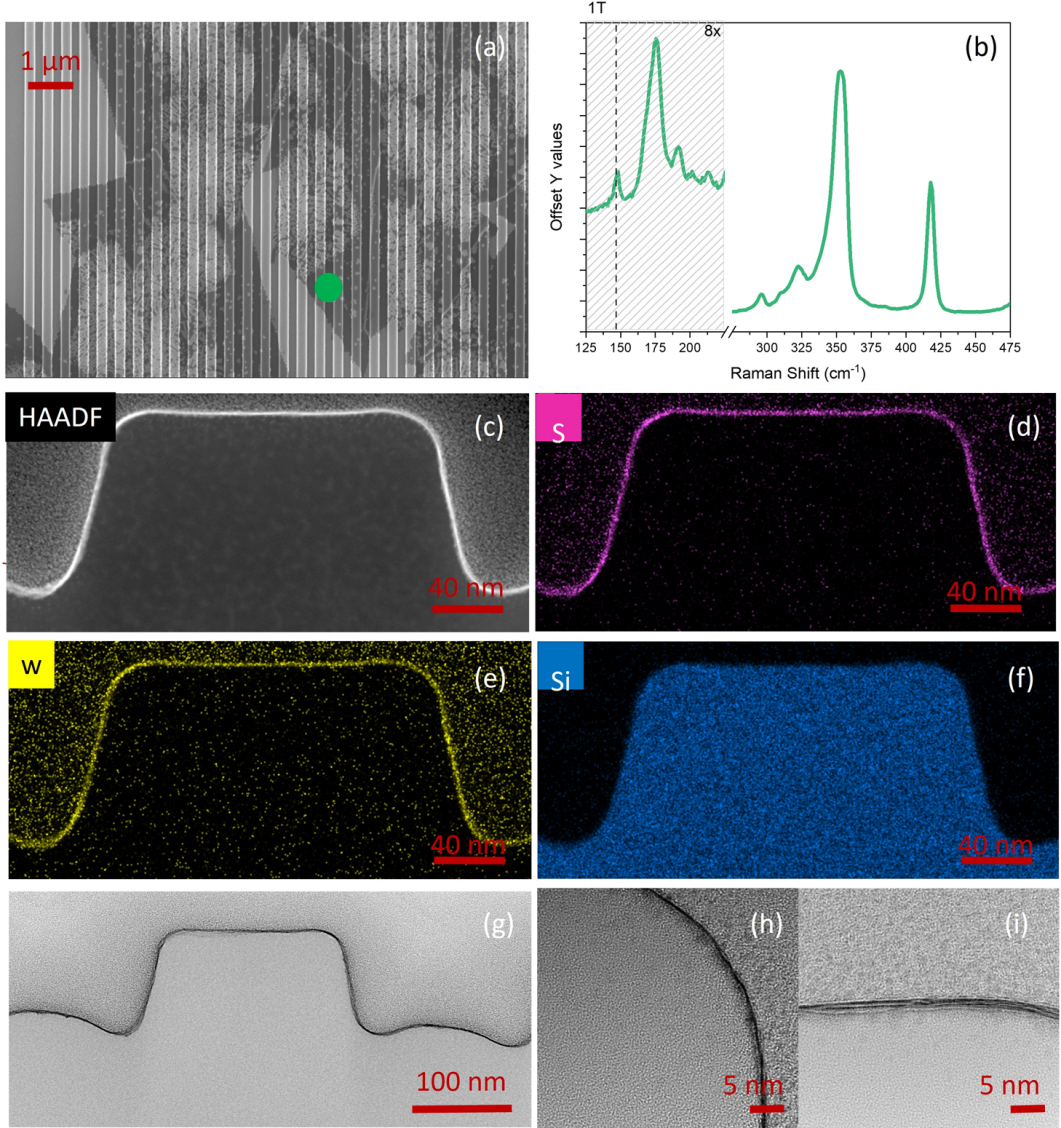
(a) Plan-view SEM image of the patterned substrate. The green dot marks the position where the Raman spectrum in (b) was acquired. For better visualization, the spectral region between 125 and 275 cm^–1^ was magnified by a factor of 8. (c) STEM image of the WS_2_ film lamella prepared from the patterned substrate shown in (a). (d,e) Corresponding EDX elemental maps of S (pink), W (yellow), and Si (blue). (f,g) Cross-sectional TEM images of the WS_2_ grown on the patterned substrate. Panels (h) and (i) show magnified views of the red and green squares in (c), respectively.

Figure 3a shows an SEM image of WS_2_ grown on the patterned substrate, where large WS_2_ flakes form a nearly continuous layer. The WS_2_ thickness is not homogeneous, with some regions appearing lighter (thicker) and others darker (thinner). The Raman spectrum acquired at the location indicated by the green dot (Figure 3b) confirms the presence of a monolayer WS_2_ as evidenced by the fit values of the E_^1^2g_–A_1g_ peak separation (62.23 ± 0.16 cm^–1^) and the 2LA/E_^1^2g_ intensity ratio (1.36 ± 0.61). Furthermore, the presence of the J_1_ peak at 147 cm^–1^ indicates that the flake is in the metallic 1T phase. We note that the effective growth of WS_2_ on patterned substrates is further demonstrated in Figure S1 in Supporting Information highlighting that the material can grow continuously and conformally across different geometries.

To investigate the degree of conformality of the WS_2_ film, cross-sectional lamellae were prepared and analyzed using aberration-corrected STEM and EDX. The interlayer spacing measured from the STEM intensity profile in Figure S2a of Supporting Information is of ∼0.60 nm and it matches well with the expected one for the WS_2_ crystal.^2^ The STEM micrographs (Figure 3c–i) show a uniform and conformal growth of the layers on the patterned Si/SiO_2_ substrate, demonstrating that the WS_2_ film adheres to the underlying geometry without delamination even at the highly curved regions of the pattern. As shown in Figure S2b,c, this is further confirmed for bending angles reaching up to 40°. Figure 3g presents a cross-sectional STEM image, while Figure 3h,i show magnified views of the red and green regions marked in Figure 3g. Both a monolayer (Figure 3h) that follows the substrate curvature, and the formation of multilayers (Figure 3i), can be observed. The chemical-sensitive EDX maps (3d–f) not only confirm the uniform distribution of W and S elements in the layers but allow for a quantitative estimation of the layer’s stoichiometry using a Brown–Powell model and assuming an estimated thickness of 70 nm (comparable with the lamella thickness used for the cross-sectional investigations of the layers) and a density of 7.5 g/cm^3^ for the hypothesized WS_2_ phase. The semiquantification was performed using the L-shell peaks of tungsten and the K-shell peaks of sulfur. The results of the analysis are summarized in Supporting Information in Figure S3a,b. For each image, an area of interest was identified and highlighted by yellow boxes, within which the semiquantitative analysis was performed. The atomic fractions extracted from the W and S elemental maps yield an atomic ratio in the range of 1:(1.8–1.9), which is in good quantitative agreement with the expected 1:2 ratio for a pure stoichiometric phase.

These complementary characterizations consistently validate the conformal growth of WS_2_ on patterned substrates, showing that the monolayer can uniformly adapt to different geometries while maintaining the 1T phase. It is interesting to observe that KCl here does not only lower the sublimation temperature of WO_3_ but also concomitantly promotes the layer adhesion on patterned substrates, in a manner similar to other seeding promoters with a more complex chemistry, such as aromatic perylene molecules, which are commonly employed for the synthesis of MoS_2_ layers on corrugated surfaces.^33^ The evidence of such improved adhesion and wettability is demonstrated by the high radii of curvature which imposes stringent adhesion requirements, as the adhesion energy between the first layer and the substrate, Γ, can be described by the relation Γ = B/R^2^,^33^ where B is the bending-stiffness of the layer and R is the radius of curvature of the patterned geometry. From this equation, it follows straightforwardly that maintaining adhesion in curved regions (with bending angles up to ∼40°, see Figure S2) without delamination requires stronger interfacial interactions between the WS_2_ layer and the substrate compared to the flat case. We therefore infer that the observed enhancement in adhesion is a secondary effect associated with the use of alkali halides. This conclusion is further supported by the observation that, in the absence of KCl, the growth of WS_2_ results in poor film formation (see Figure S4 in Supporting Information).

### WS_2_ Stability under Ambient and Thermal Conditions

As the 1T phase of WS_2_ is metastable,^10,34^ its long-term stability should be evaluated. The aging of monolayer 1T-WS_2_ was monitored using Raman spectroscopy on both flat and patterned substrates. The samples were stored in a drybox under a continuous nitrogen flux for approximately two months. During this period, they were periodically analyzed and thus periodically exposed to light and ambient conditions (temperature and humidity of 21 °C and 40%, respectively). Figure 4a presents the Raman spectra of WS_2_ monolayers grown on flat and patterned substrates, collected immediately after synthesis and after one month of storage. Spectra were acquired from multiple spots across different monolayers. No significant spectral differences were observed between freshly prepared and aged samples. Notably, the characteristic J_1_ mode remains clearly visible, indicating that no degradation of the 1T phase occurred over time.

**Figure 4:**
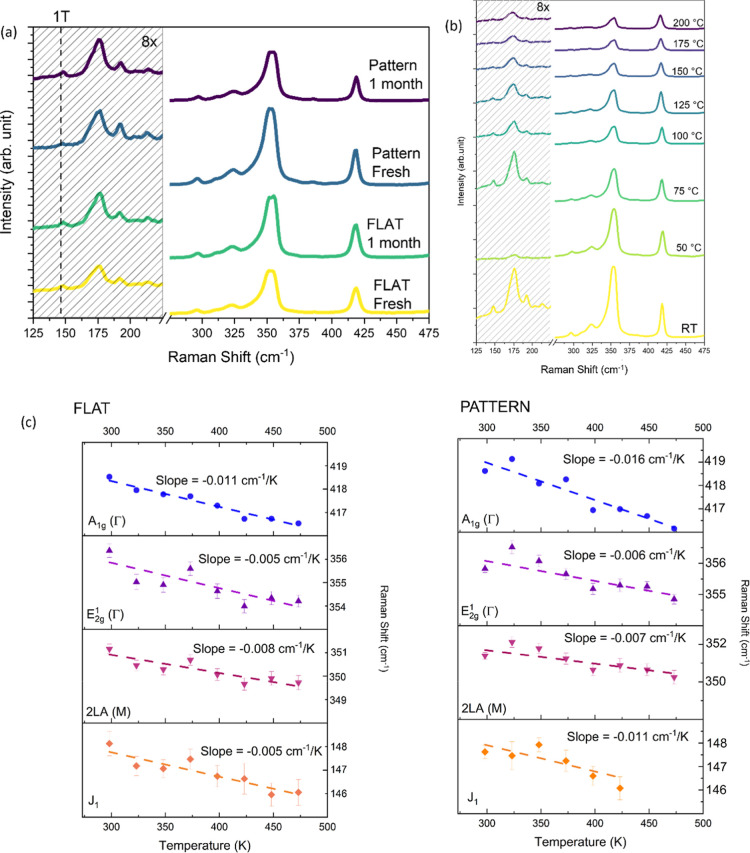
(a) Aging of the WS_2_ grown on flat and patterned substrates studied by Raman investigations conducted on fresh samples and after 2 months. (b) Representative Raman spectra of WS_2_ growth on patterned substrate at varying temperatures. (c) Temperature dependence of Raman frequencies of A_1g_, E^1^_2g_, 2LA, and J_1_ modes, respectively. The dash lines represent the outcomes of linear fit.

Even the thermal stability of 1T-phase WS_2_ monolayers was investigated on both flat and patterned substrates. Figure 4b displays temperature-dependent Raman spectra of WS_2_ grown on a patterned substrate, measured over a temperature range from room temperature to 200 °C. As the temperature increases, the positions of the A_1g_, E_^1^2g_, and 2LA modes gradually shift toward lower frequencies. The temperature dependence of the A_1g_, E_^1^2g_, and 2LA spectral positions was analyzed using a linear approximation based on the equation^35^

1 $$\omega \left(T\right)=\omega_{0}+\chi T$$

where ω_0_ is the extrapolated Raman shift at 0 K, and χ is the first-order temperature coefficient. The extracted χ values for each vibrational mode were reported in Figure 4c.

By comparison between the flat and patterned configurations, there is a slight difference in the extracted temperature coefficients. In both cases, the Raman modes redshift with increasing temperature due to anharmonic phonon interactions and softening of force constants. The slopes extracted from the linear fitting defining the χ value for the flat (patterned) configurations are −0.011 (−0.016) and −0.005 (−0.006) cm^–1^ K^–1^ for the A_1g_ and E^1^_2g_ modes, respectively. Our results are in good agreement with values reported in ref 36 where the dominant contribution to the temperature-dependent Raman shift of the in-plane E^1^_2g_ mode is attributed to three-phonon anharmonic processes, while thermal expansion provides the primary contribution to the shift of the out-of-plane A_1g_ mode. This behavior aligns with the Grüneisen constant model, which predicts that volume expansion induces larger Raman shifts in low-degeneracy modes like A_1g_ (degeneracy, *n* = 1) compared to higher-degeneracy modes like E^1^_2g_ (*n* = 2).^37^ Even the low-frequency modes, 2LA and J_1_, show a linear dependence in the temperature range considered here. For the flat and patterned configurations, the extracted χ values are −0.008 (−0.007) cm^–1^ K^–1^ for the 2LA mode and −0.005 (−0.011) cm^–1^ K^–1^ for the J_1_ mode. It should be noted that, for the highest temperatures considered, the J_1_ peak in the patterned sample is barely visible (see Figure 4b), making it challenging to reliably fit the curve and determine its frequency position. For this reason, the temperature dependence of this mode in the patterned sample is extracted only for temperatures below 150 °C (Figure 4c). Notably, upon cooling down at room temperature, J_1_ intensity is recovered demonstrating the thermal stability of the 1T-WS_2_ phase (Figure S5 in Supporting Information). The substantial similarity of the extracted χ values for the flat and the considered patterned configurations can be explained by the negligible role of the substrate on the temperature coefficients of the Raman modes, as observed by comparing supported and suspended WS_2_ flakes.^38^

## Conclusion

In summary, we demonstrated the successful synthesis of large-area 1T-phase WS_2_ (up to 100 μm) monolayers by optimizing the salt-assisted CVD process. Introducing the KCl as alkali metal halide, it is possible to obtain thermally stable (up to 200 °C) single crystal 1T-WS_2_ onto flat and patterned substrates, thus demonstrating a conformal growth. Overall, these results highlight a reproducible pathway to obtain large-area, conformal, and stable 1T-WS_2_ monolayers. This approach not only provides new insights into phase engineering in TMDs but also paves the way for the integration of metastable 1T-WS_2_ into device architectures requiring uniform coverage on nonplanar substrates.

## Supplementary Material



## Notes

Additional SEM, TEM, and EDX micrographs of the layers grown on patterned substrates; and additional experimental details and Raman spectra of the samples (PDF)
