# Supplementary material for: Conformal Growth of Stable 1T-Phase WS2 by Alkali Metal-Halide-Assisted Chemical Vapor Deposition
Source: Cryst Growth Des. 2026 Jul 18;26(15):5905–12. doi: 10.1021/acs.cgd.6c00304 (PMC13455585; doi:10.1021/acs.cgd.6c00304)
Supplement: Supplementary file 1 [file cg6c00304_si_001.pdf]

Supporting Information

Conformal Growth of Stable 1T-Phase WS_2 by Alkali Metal-Halide-Assisted Chemical Vapour Deposition

Alessia Motta,[1] Andrea Serafini,[2] Paolo Targa,[2] Davide Codegoni,[2] Carlo Grazianetti,[1*] and Christian Martella[1*]

[1] CNR IMM, Unit of Agrate Brianza, Via C. Olivetti 2, I-20864 Agrate Brianza, Italy

[2] STMicroelectronics, Via C. Olivetti 2, I-20864 Agrate Brianza, Italy

*carlo.grazianetti@cnr.it (CG), christian.martella@cnr.it (CM)

Keywords: tungsten disulfide; chemical vapor deposition; alkali metal halide; shape engineering; seeding promoter;

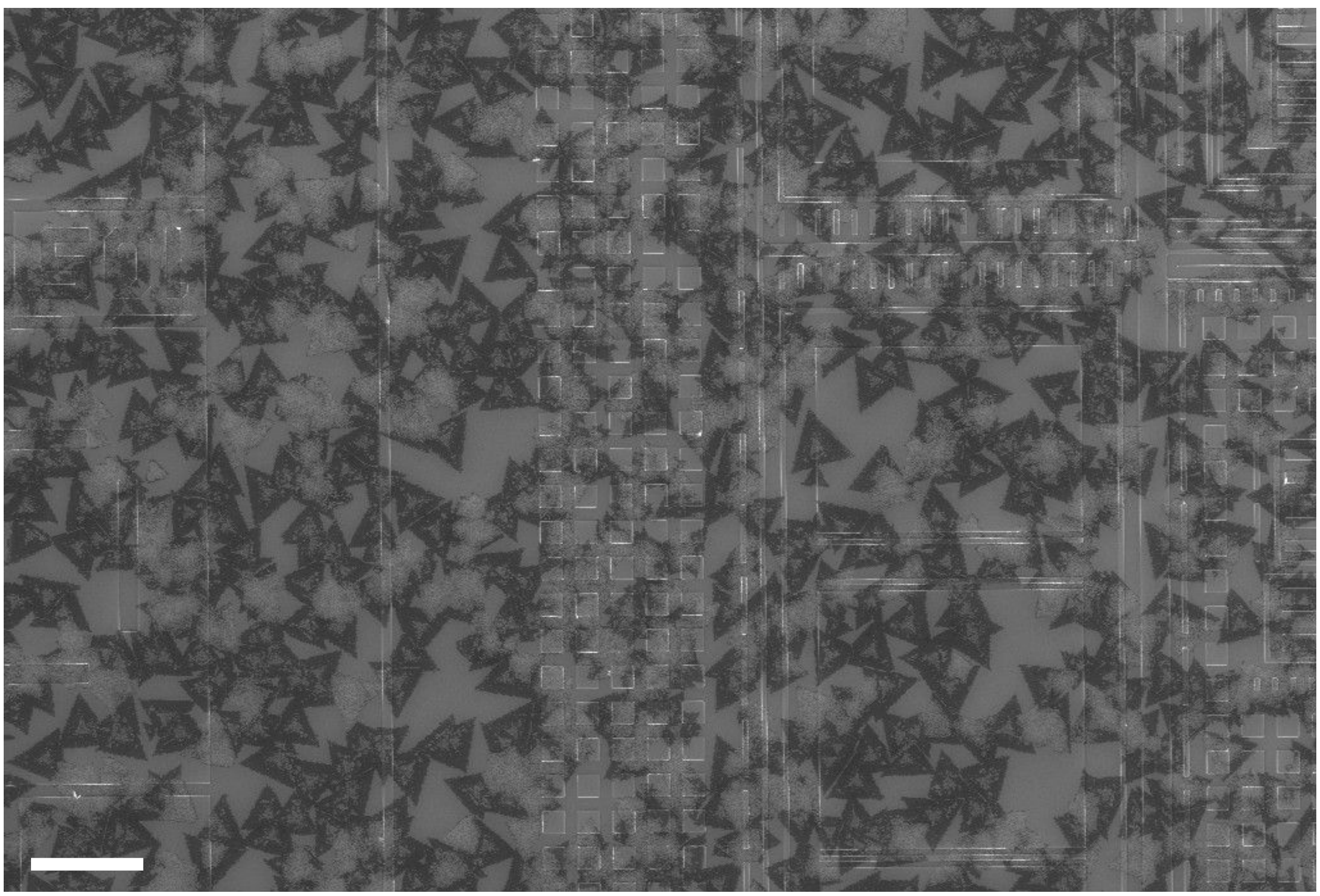

Figure S1. SEM image showing the conformal growth of the WS_2 layers on different substrate patterns. Scale bar is 10 µm.

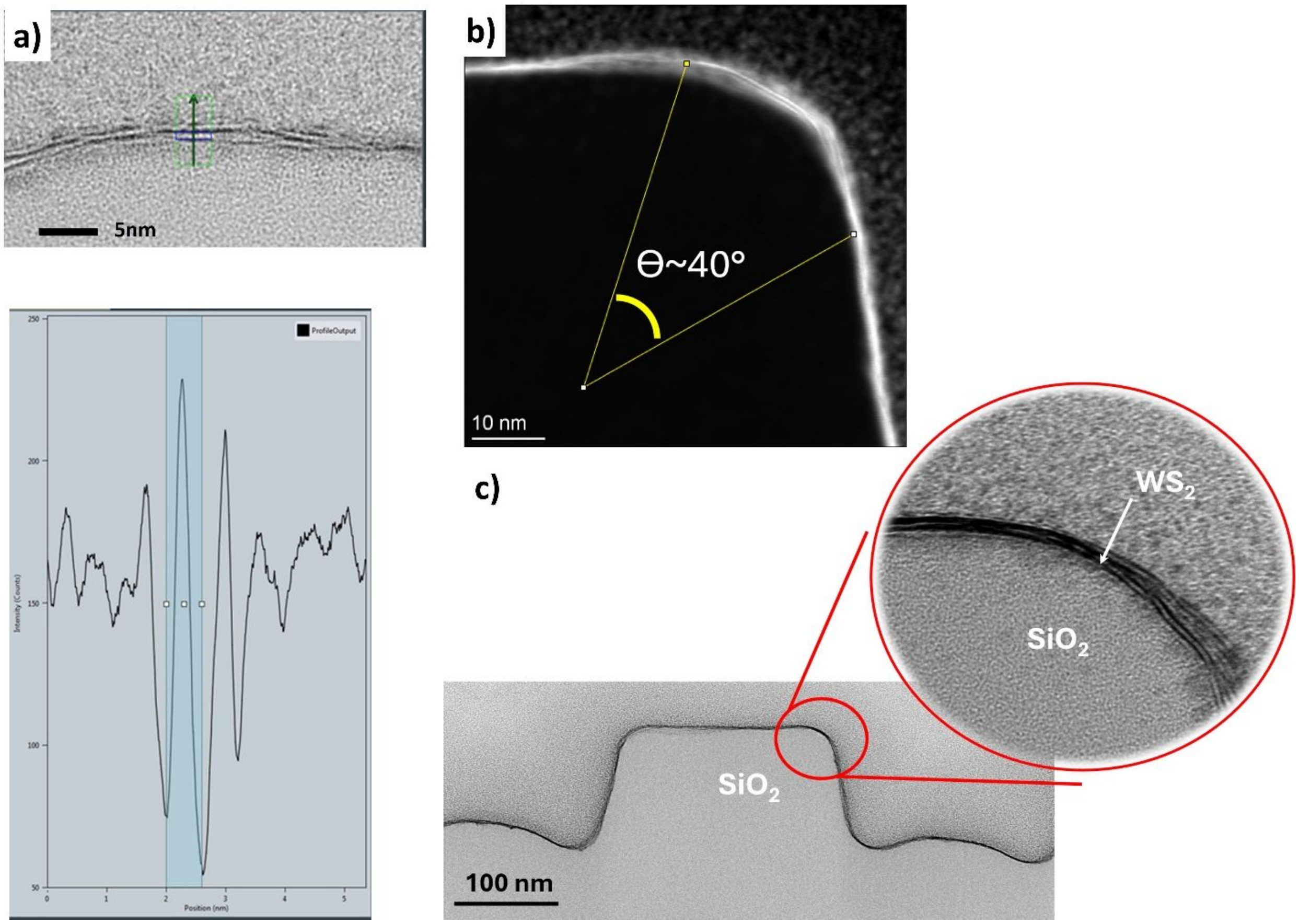

Figure S2. (a) Interlayer spacing measured by STEM (top) intensity profile (bottom). (b) HAADF TEM cross-sectional micrographs showing conformality of the layers at bending angles as high as ~40°. (c) Cross-sectional STEM image confirming that the layers adhere well to the substrates, with no evidence of delamination, even at the curved regions of the patterned structures.

a)

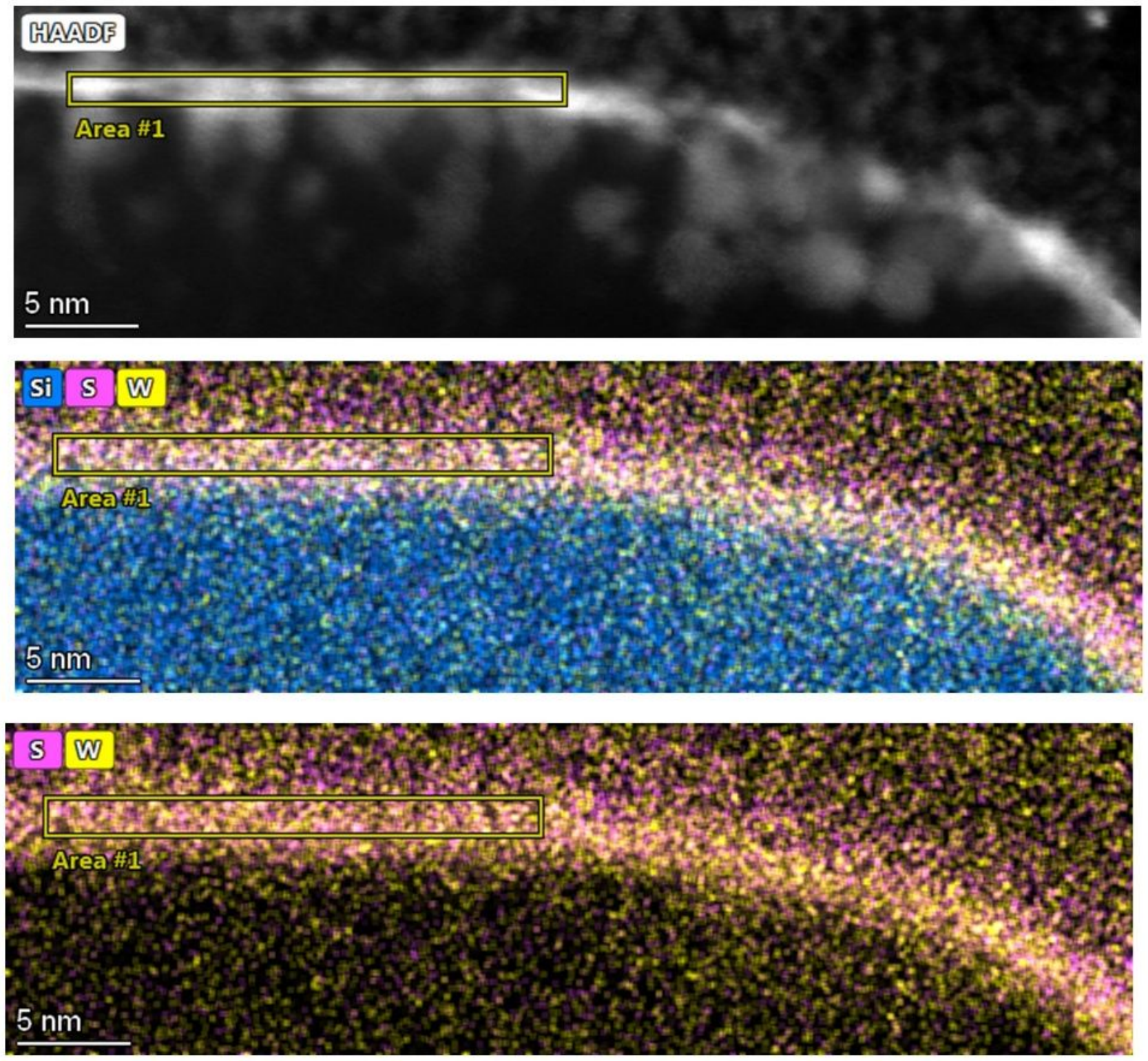

Z	Element	Family	Atomic Fraction (%)	Atomic Error (%)	Mass Fraction (%)	Mass Error (%)	Fit error (%)
16	S	K	65.54	8.39	24.91	2.19	2.89
74	W	L	34.46	5.70	75.09	10.27	0.29

b)

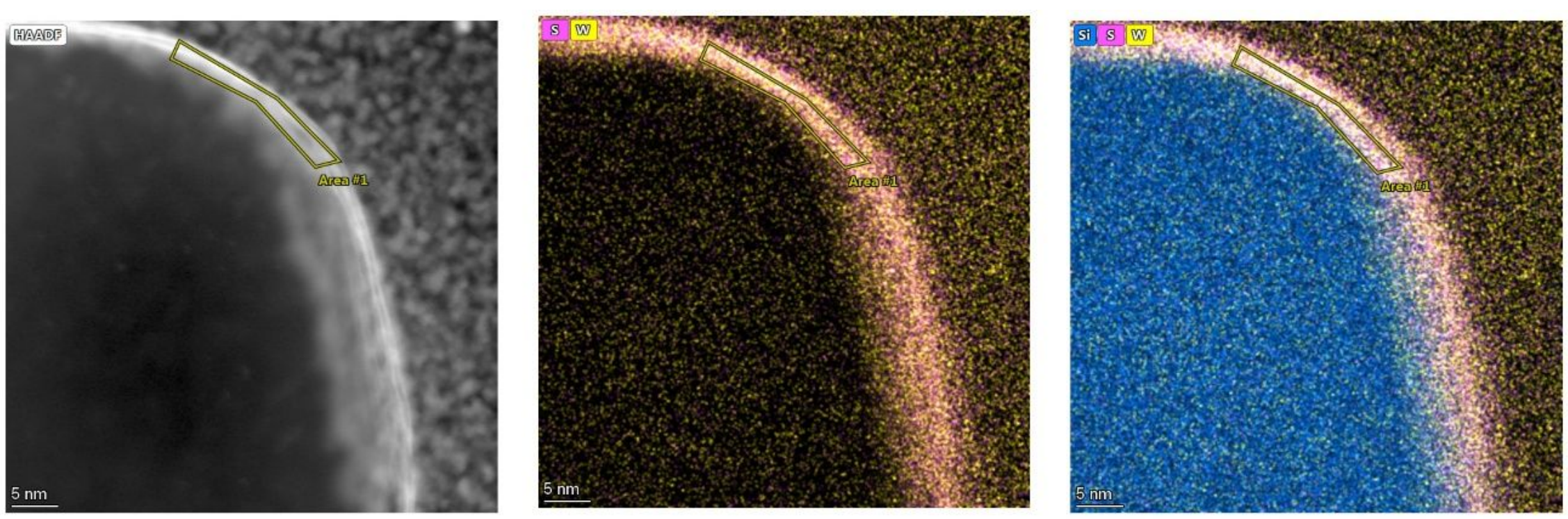

Z	Element	Family	Atomic Fraction (%)	Atomic Error (%)	Mass Fraction (%)	Mass Error (%)	Fit error (%)
16	S	K	64.30	8.10	23.91	2.04	1.46
74	W	L	35.70	5.91	76.09	10.45	0.21

Figure S3.(a)–(b) Atomic fractions of W and S extracted from the EDX maps. The derived W:S ratio in the range of 1:(1.8–1.9) is in good quantitative agreement with the expected 1:2 ratio for a pure stoichiometric phase.

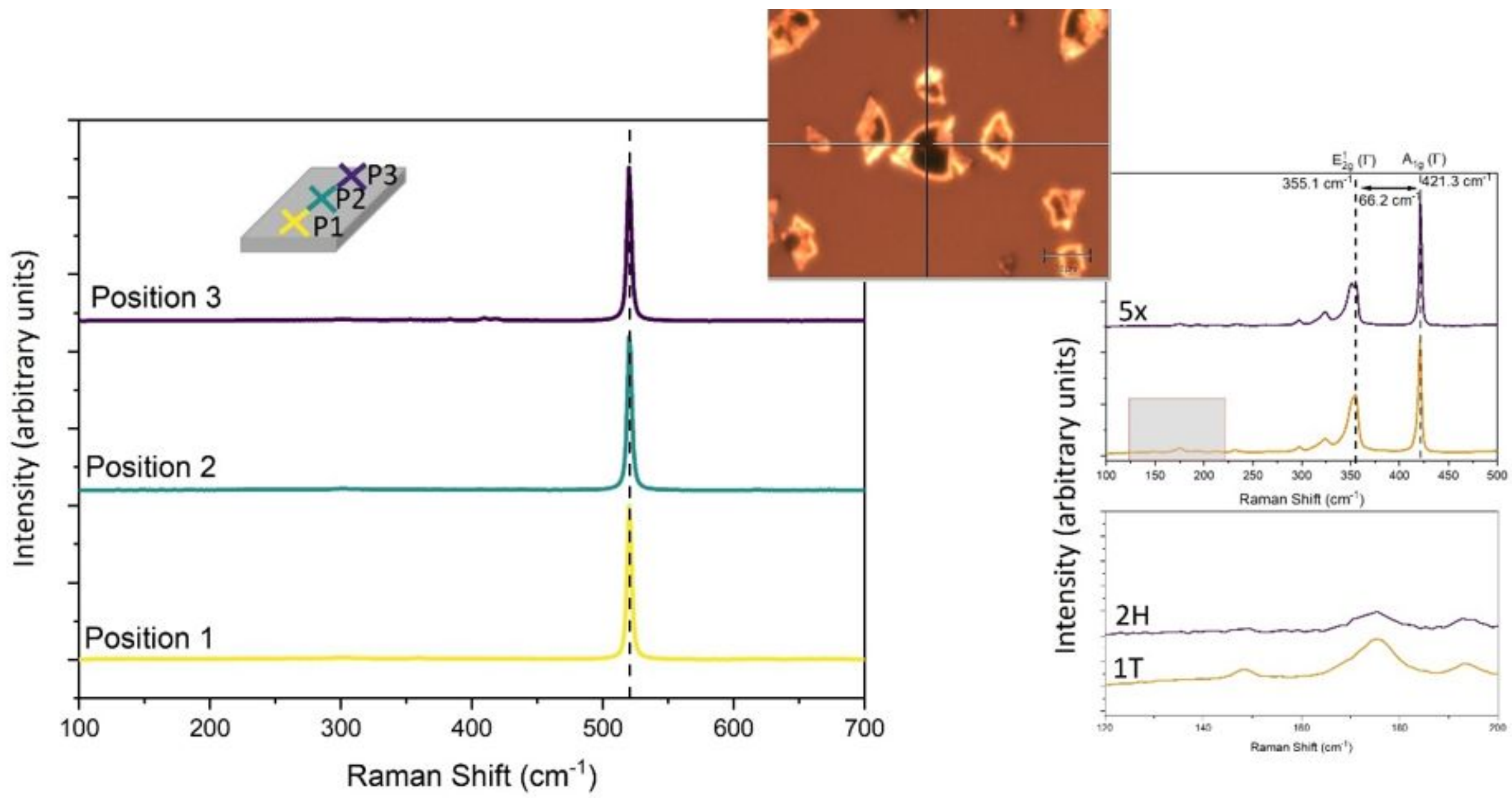

Figure S4. Growth of WS_2 without KCl promoter. Raman spectra acquired at three spatially separated positions (P1, P2, and P3, 1 cm apart) across the sample show very weak WS_2 signals in the 100–500 cm^{-1} region, with the spectra dominated by the Si peak at 520 cm^{-1}. The inset optical image (scale bar: 10 μm) highlights the sparse, cluster-like morphology, confirming the low spatial density and poor film continuity in the absence of alkali halide promoters. A closer inspection of the 100–500 cm^{-1} region reveals only low-intense WS_2 vibrational modes, characterized by the presence of a mixed 1T–2H phase with low crystallinity and limited surface coverage.

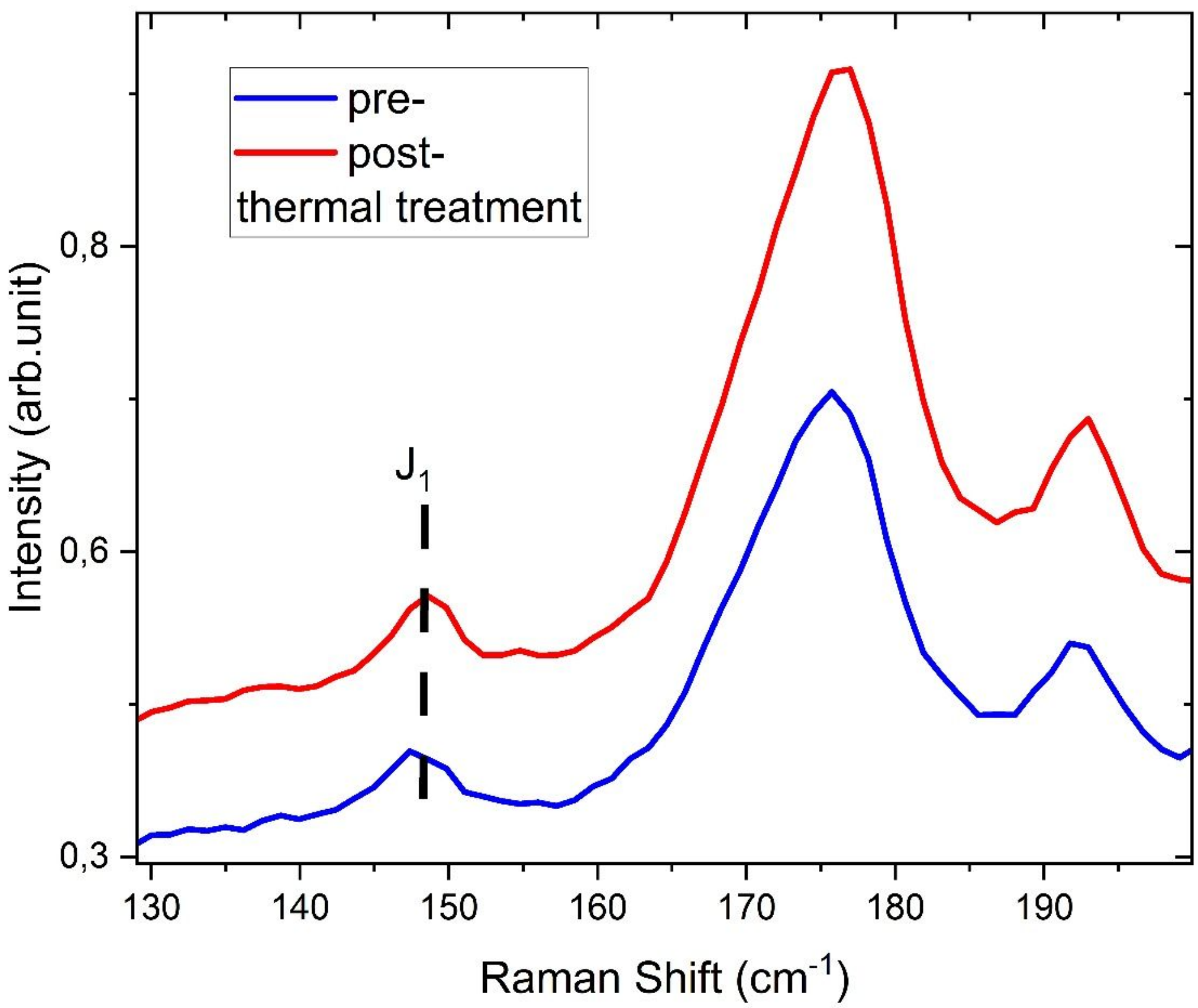

Figure S5. Raman spectra of WS_2 in the 130–200 cm^{-1} frequency range were acquired at room temperature before and after thermal treatment up to 200 °C. The two spectra are vertically offset for clarity. The J_1 peak associated with the 1T phase is clearly visible both before and after the treatment.
